# Evaluating Nondestructive Quantification of Composition Gradients in Metal–Organic Frameworks by MeV Ion Microbeam Analysis

**DOI:** 10.1021/acs.analchem.4c02730

**Published:** 2024-09-10

**Authors:** Gyula Nagy, Wanja Gschwind, Sascha Ott, Daniel Primetzhofer

**Affiliations:** † Department of Physics and Astronomy, Uppsala University, SE-751 20 Uppsala, Sweden; ‡ Department of Chemistry–Ångström Laboratory, Uppsala University, SE-751 20 Uppsala, Sweden

## Abstract

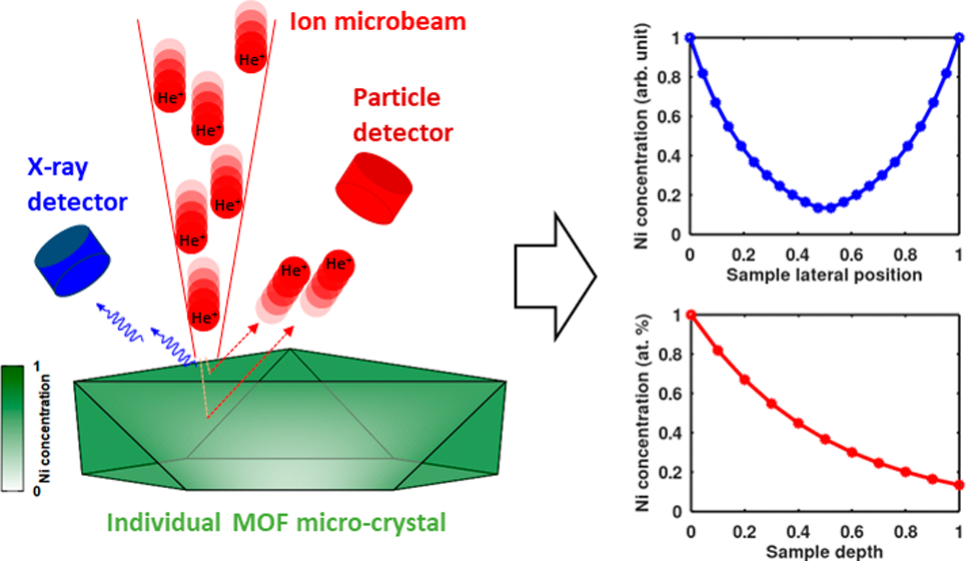

We evaluate a method to quantify composition depth gradients in intact metal–organic framework (MOF) single crystals and thereby derive diffusion coefficients of postsynthetically incorporated active sites by nondestructive ion-beam microanalysis. Zr-based UiO-67-bpy (bpy = 2,2′-bipyridine-5,5′-dicarboxylic acid) MOFs were synthesized on Si substrates and then metalated postsynthetically with NiCl_2_ for 2–48 h, resulting in different Ni depth distributions. Simultaneous micro-Rutherford backscattering spectrometry (μ-RBS) and micro-particle induced X-ray emission (μ-PIXE) analysis were used for the spatially resolved chemical analysis of the MOF single crystals. Qualitative assessment of the μ-RBS spectra indicated the presence of elemental depth gradients and hinted at the governing process of the postsynthetic Ni incorporation, in the present case, molecular diffusion. Quantitative evaluation of the resulting composition depth profiles directly provided the diffusion length and, thereby, the diffusion coefficient of the system. Virtual gradients caused by overhanging tips/edges of the truncated octahedral crystal shape are considered. Furthermore, in the case of insufficient probing depth for μ-RBS, μ-PIXE was still capable of providing qualitative information. In the present system the diffusion coefficient for NiCl_2_ is found to be (1.72 ± 0.18) × 10^–16^ m^2^s^–1^. The long-term stability of the synthesized and postsynthetically modified MOFs is proved by repeated measurements.

## Introduction

Metal–organic frameworks (MOFs) feature an extremely large surface to volume ratio, making them highly attractive in gas capture,^1^ catalysis,^2−5^ molecular separation,^6^ etc. The tunability of MOFs originates from their multicomponent nature, where inorganic clusters, so-called secondary building units (SBUs) are interconnected via organic linkers which can host a number of functional groups that define their properties.^7−10^ Active sites are often introduced postsynthetically into present metal binding sites. In other words, the MOFs are first synthesized from their building blocks to form crystallites into which additional reagents are introduced, while preserving the lattice structure.^11−13^ Intentional spatial modulation of these active sites gives an enormous potential to fabricate tailor-made MOFs, and the core–shell pattern is a particularly promising direction in MOFs engineering, as outlined in ref (12). Since then, such core–shell structures have indeed been proven to be superior in their functionality compared to that of MOFs with uniform composition in some instances. For example, deactivation of the catalytic sites on the MOF surface was shown to enhance size-selective reactivity,^14^ while heterojunction structures improve photocatalytic performance.^15,16^ Furthermore, hollow multishell MOFs^17,18^ further diversify the possibilities by providing a hierarchical scaffold for catalytic sites.^19^

However, during the development of new MOF structures, the spatial distribution of the constituents in the final MOF structure is usually unknown,^20^ and suitable analysis techniques are required to sensitively probe the final composition of a MOF. Several analytical techniques have been successfully employed to characterize the structure of MOFs, each having their own strengths and weaknesses. In the case of core–shell heterostructures, the composite nature results in distinct crystallographic and morphological/microstructural properties between the core and the shell regions, and thus X-ray diffraction (XRD),^15^ transmission electron microscopy (TEM)^17,18^ or scanning electron microscopy^21^ might be used to reveal the core–shell fashion of the created MOF. For a bimetallic MOF based on a single crystalline structure, TEM combined with energy-dispersive X-ray (EDX) mapping distinguished the different metallic sites,^22^ although rather qualitatively. Furthermore, when the molecules/fragments can be uniquely discriminated by mass, matrix-assisted laser desorption ionization time-of-flight (MALDI-TOF) mass spectrometry can be utilized,^23^ a process that is however destructive to the MOF. Additional techniques used to analyze the spatial element structure, without claim of being exhaustive, are micro-Raman spectroscopy,^14,24^ photothermal induced resonance,^25^ confocal fluorescence microscopy,^26^ X-ray photoelectron spectroscopy (XPS),^27^ and Rutherford backscattering spectrometry (RBS).^28−30^

Micro-Rutherford backscattering spectrometry (μ-RBS) is a versatile, nondestructive ion-beam analytical method that can provide laterally and depth-resolved information on the elemental composition of a micrometer-sized sample. A decaying content of an element along the sample depth shows up in the RBS spectrum as a decaying signal intensity toward lower backscattered ion energies of this particular element. This information, however, cannot be directly transformed to an absolute concentration gradient value because (i) backscattering cross sections change nonequally with projectile energy for different target atom masses and (ii) detected backscattered projectile energies correspond to different sample depths for the different target masses. We have already shown in a previous work that μ-RBS is a unique tool for the sensitive, nondestructive elemental depth profiling of intact MOF single crystals, and we showed that the technique allows to distinguish between core–shell and uniform distribution.^31^

In the present work, we propose a method for the quantification of the composition depth gradients using μ-RBS and apply the method to UiO-67 type MOF single crystals with incorporated bipyridine metal chelators that are metalated with NiCl_2_. In this system, and in contrast to earlier works, the elements of interest, in particular Ni, are not the heaviest constituents of the sample, i.e., their signals in the RBS spectrum are not clearly separated from that of the heavier matrix. In addition, we show a special case when RBS is limited because of its information depth, but simultaneous particle-induced X-ray emission (PIXE) analysis can still provide valuable information regarding the spatial distribution of the elements in the sample in a qualitative manner.

## Experimental Section

### MOF Synthesis and Postsynthetic Metalation

**Figure 1:**
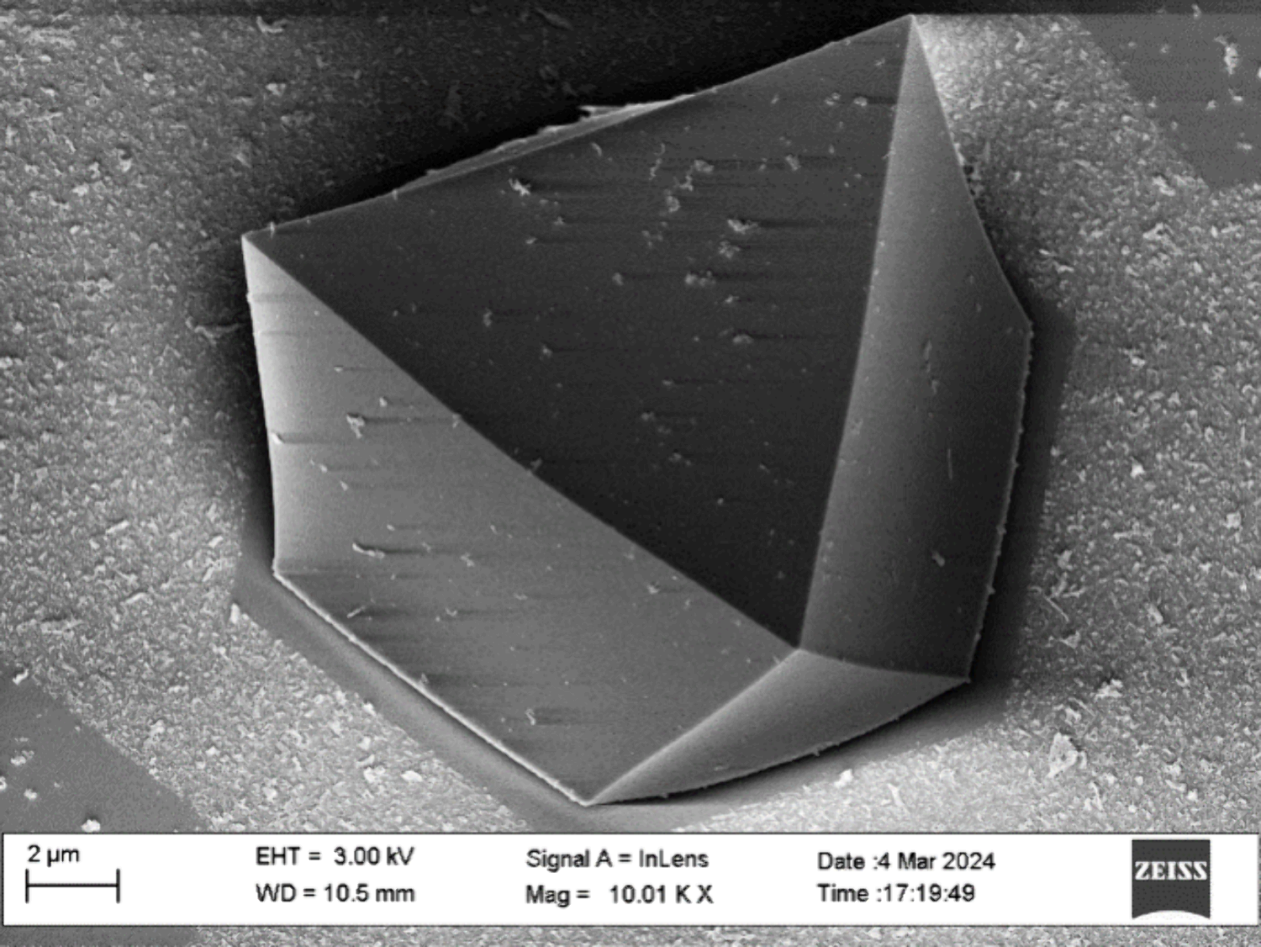
Typical SEM image of a UiO-67-bpy MOF single crystal as analyzed in this study, commonly oriented along the (111) plane.

Zr-based mixed-linker UiO-67-bpy (bpy = 2,2′-bipyridine-5,5′-dicarboxylic acid) MOFs, containing approximately 30% bpy linker and 70% biphenyl-4,4′-dicarboxylic acid (bpdc) linker, were synthesized on Si substrates following the protocol reported in ref (31) and then metalated with NiCl_2_ at 100 °C for 2–48 h. The linker ratio was selected according to our recent results that showed the linker ratio strongly influences the diffusion speed:^32^ UiO-67 MOFs with 30% bpy-to-bpdc ratio and metalated for 2–48 h were found to exhibit characteristic features in the RBS and PIXE spectra that are relevant to this work. One sample typically contained some hundreds of MOF crystals, and the size of individual crystallites on the Si substrate was found to range from ca. 15–20 μm. A typical SEM image is presented in Figure 1, illustrating a single MOF crystallite oriented along the (111) plane, as used later in this study for elemental depth-profiling. Optical microscope images can be found in the Supporting Information (see Figures S1 and S2).

The crystallinity of the synthesized MOFs was analyzed by X-ray diffraction (XRD). The resulting XRD pattern (see Figure S3) reproducibly featured only the <111> and <222> reflections, which aligned well with the respective reflections of the simulated XRD pattern of UiO-67. Additionally, the successful creation of the mixed-linker nature of our MOFs was confirmed by nuclear magnetic resonance (NMR) measurement (see Figure S4). The bpy-to-bpdc ratio was calculated from the NMR spectrum to be 30 ± 3%.

### Ion-Beam Microanalysis

After the metalation process, the chemical composition of selected individual crystals which are oriented along the (111) plane, was analyzed by simultaneous μ-RBS and μ-PIXE at the scanning nuclear microprobe^33^ in the Tandem Laboratory, Uppsala University.^34^ A beam, composed of 5 MeV He^2+^ ions and focused to a spot of 3–4 μm, was used. The large divergence of the beam minimized the possibility of signal loss due to channeling effects in the crystalline sample. The intensity of the beam was ca. 50–150 pA. It was shown in ref (31) that, due to the large power density of the microfocused ion beam, a stationary beam may cause damage to the MOF crystallites, and it is also known that even in the case of insufficient scanning speed the thermal load of the sample may be substantial.^35^ In order to avoid any undesired side effects, the beam was scanned over the entire crystallites with a properly chosen scanning speed on the order of 500 μm/s. Optical and scanning electron microscope images taken after the measurement showed no evidence of any detectable damage caused to the MOFs.

During the measurements, the energy of the backscattered projectile ions was detected using an annular-type Si solid state particle detector and digitalized by an MPA-3 data acquisition system (FAST ComTec GmbH), resulting in μ-RBS spectra (i.e., counts vs backscattered ion energy) on the data acquisition PC. Simultaneously to the detection of the backscattered projectiles, characteristic X-rays induced by the incident beam were detected by means of a Si(Li) X-ray detector placed at 135° relative to the incident beam direction. μ-PIXE spectra display counts vs X-ray photon energy. In the present configuration, X-ray energies above that of Si K_α_ can be detected, i.e., Zr, as a part of the base MOF structure and Cl and Ni, as postsynthetically incorporated elements.

## Discussion

First, two samples were separately metalated with NiCl_2_ for 2 and 24 h. During the metalation process, NiCl_2_ diffuses from the surrounding solution into the MOF pores where a reaction takes place, during which Ni^2+^ coordinates to the bipyridine-based linkers. The final spatial distribution of Ni^2+^ is determined by competing processes. In essence, if the Ni^2+^ diffusion is faster than the reaction with bpy, one may expect a nearly equal amount of Ni everywhere throughout the crystal, and a resulting uniform or random distribution is expected. In the case of much slower diffusion compared to the Ni coordination rate, the resulting distribution is expected to show a core–shell pattern with a Ni concentration gradient from the outer surface toward the crystal core. If diffusion speed and coordination speed are comparable, then the final distribution of Ni is described by a superposition of the two competing processes. As a completely different scenario, in the case of preferential reaction on the surface due to enthalpic penalty,^24^ the result is near-surface distribution. An additional complication is that Ni^2+^ cations that diffuse into the crystal at a later time point of the PSM experience a different chemical environment with smaller pore apertures due to already incorporated Ni centers than those at earlier times. This difference in microenvironments may lead to nonlinear diffusion coefficients.

### Element Maps

**Figure 2:**
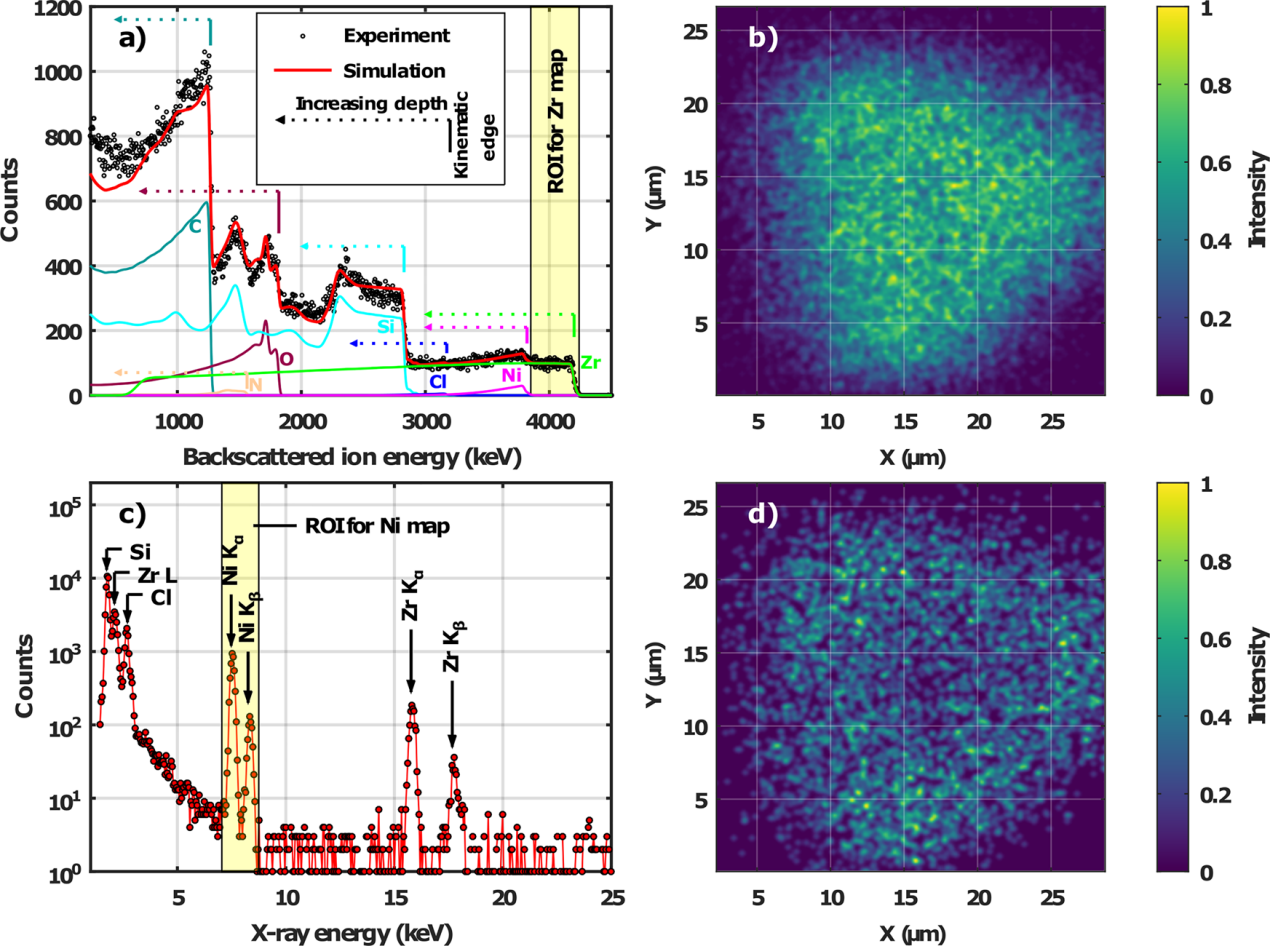
(a) μ-RBS spectrum showing simulated results with individual element contributions, (b) Zr μ-RBS map, (c) μ-PIXE spectrum, and (d) Ni μ-PIXE map of a MOF single crystal metalated for 2 h.

Both μ-RBS and μ-PIXE are capable of providing 2D element maps based on selected regions of interest in the corresponding energy spectra. Element maps are built up by assigning the physical coordinates of the ion microbeam to the spectrum counts by coincidence recording of the scan controlling signals and the signals originating from the detectors. Figure 2 illustrates the RBS and PIXE spectra and maps of the MOF crystal metalated for 2 h. Data acquisition is list-mode, meaning that the element maps and basically arbitrary cuts can be extracted from the collected data during postprocessing, the importance of which is addressed later. μ-PIXE by its nature provides characteristic peaks for individual elements, and if a peak is not overlapping with another one nor affected by artifacts or background, the element concentration is proportional to the number of counts in a pixel. If peaks are overlapping with each other, a spectrum deconvolution method such as the dynamic analysis^36,37^ is necessary. In contrast, μ-RBS measures the energy of the backscattered projectiles, which carries information about the mass of the backscattering element, as well as its depth within the matrix. As a consequence, the signal of lighter elements that are closer to the surface is superimposed on the signal of heavier elements that are deeper inside the material (see Figure 2a for visual explanation: the kinematic edge of each element corresponds to a surface element). In addition, for lighter elements, nuclear resonances further complicate the spectrum through energy-dependent, resonant backscattering cross sections (see the Si, O, N and C signal in Figure 2a). Advanced RBS simulation software packages are capable of taking all these features into account by considering in their calculations (i) electronic stopping power data providing quantitative depth perception, and (ii) measured or evaluated elastic backscattering cross sections, providing concentration quantification. However, this complication appears also in the element maps, and although efforts are being made to extend the dynamic analysis to RBS spectra,^38^ to-date no RBS simulation software exists which is capable of building element maps by proper decomposition of the RBS signal. Therefore, considering unprocessed RBS spectra, the only representative element map is the one corresponding to the highest backscattered energy region and only above the next signal that appears in the spectrum, in our case, Zr (see the region of interest in Figure 2a). The μ-PIXE spectrum of the postsynthetically metalated UiO-67-bpy MOF, illustrated in Figure 2c, shows a clean sample with no detectable contaminants (note that Si originates from the substrate under and around the MOF crystal). The element maps (Figure 2b,d), built up on the selected regions of interest as indicated by the yellow rectangles in the RBS and PIXE spectra, correspond to the Zr lateral distribution on the top ≈1.7 μm and the Ni distribution through the full crystal thickness, respectively. From these maps, it turns out that Zr is homogeneously distributed across the crystal, while Ni is more concentrated at the edges, indicative of a core–shell pattern.

### Quantification of Depth Gradients

The recorded μ-RBS spectra were processed using the SIMNRA simulation software.^39^ Our constructed model incorporates several features to obtain accurate information on the composition of the MOFs. In order to account for the composition depth gradient, a multisliced target was used in the simulations. The elements of particular interest for the target, namely Ni and Cl, featured a gradient according to a hypothetical gradient function, while the nongradient elements, i.e., those that are part of the base MOF structure, had constant element fractions throughout the whole target thickness according to the nominal composition of UiO-67-bpy MOF. Specifically, every SBU metal cluster is assumed to connect to 6 linkers, 30% of which (1.8 per Zr cluster on average) is bpy while the rest is bpdc, resulting in a nominal atomic concentration summarized in Table 1. Note that H is not visible in the RBS spectrum due to the kinematically forbidden backscattering reaction of He from H. Still, it contributes to the electronic stopping, which is considered in the SIMNRA simulations.

**Table 1:** Nominal Atomic Composition of the UiO-67-bpy (30% Bpy Linker) MOF Crystal before Metalation, as Used in the SIMNRA Simulations; after Metalation, the Same Ratio Is Maintained between These Elements

element	concentration (atom %)
H	33.11
C	44.08
N	1.97
O	17.54
Zr	3.29

Assuming Fickian diffusion from an inexhaustible source, based on the assumption that the solvent concentration does not change significantly during the metalation process, the time-dependent depth distribution of the diffusant follows the equation
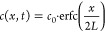
1where *c*_0_ is the surface concentration, *x* is the depth, and *L* is a characteristic diffusion length, where the concentration drops to . The diffusion length can be expressed as
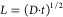
2where *t* is the elapsed (metalation) time and *D* is the diffusion coefficient. Visual inspection of the raw RBS spectrum (see the Ni signal in Figure 2a) supports a similar function shape, i.e., a function with a first derivative raising from negative values at the surface asymptotically toward zero inside the bulk, implying that diffusion is indeed the governing process in the investigated system. Therefore, in the following, the composition gradient is described using the complementary error function as presented in eq 1.

**Figure 3:**
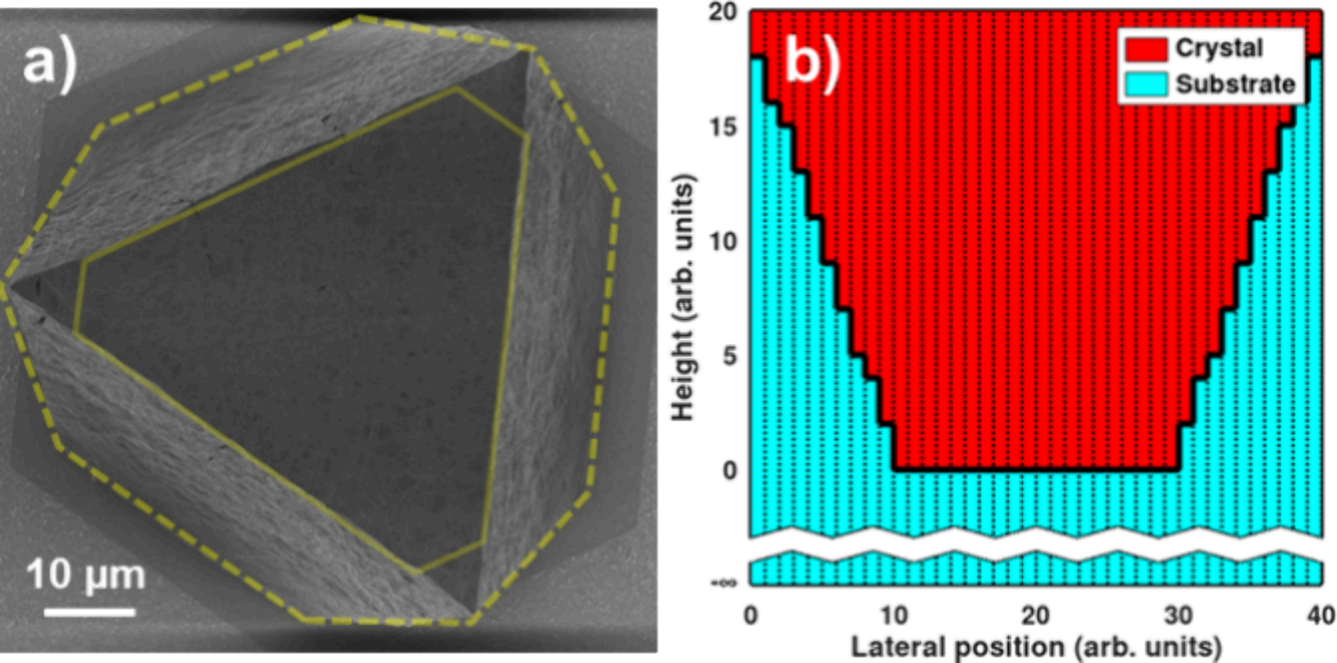
(a) SEM image of a MOF single crystal, illustrating area measurements for the parameters used in eq 3. The area within the solid line defines *A*_top_, while the dashed line defines *A*_bottom_. (b) Equivalent shape (2D cross section), consisting of multiple vertical slices with different crystal thicknesses above the thick substrate, as used in our RBS simulation model.

In addition to the true element gradients, overhanging tips and edges as a consequence of the truncated octahedral crystal shape result in a virtual gradient in the RBS measurement for all elements. The reason for these virtual gradients is that the effective thickness of the crystal is gradually decreasing toward its edges, resulting in an elevated near-surface contribution in the spectrum for each element, including the base crystal constituents. In order to account for this effect, a shape correction factor was introduced into our model, defined as the relative area occupied by the overhanging regions. In the RBS simulations, it was considered by a linear transition from zero to maximum crystal thickness, but since only a finite number of function evaluations is possible during the spectrum fitting procedure, the transition linear was approximated by a step-function. Specifically, the target was vertically sliced, and the slices representing the edges corresponded to different target thicknesses above the Si substrate. The cumulative simulated spectrum was built up by the algebraic sum of the spectra corresponding to individual slices. The relative area occupied by the overhanging edges, according to scanning electron microscope (SEM) measurements illustrated in Figure 3a, is
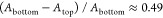
3and the visual representation of such a shape is shown in Figure 3b. However, this approximation holds true only in the case of a thin sample, i.e., when the full crystal thickness is probed by the beam. Furthermore, potential crystal defects might also result in a similar virtual gradient effect. Hence, the calculated shape correction factor served only as an initial estimate, and in the RBS simulation model, it was treated as a free parameter.

In order to find the model parameters (surface concentrations and gradient values) of the gradient elements as well as the shape correction factor, the deviation between the measured and simulated spectrum was minimized. It was carried out by defining a cost function on the goodness of the fit, denoted as χ_red_^2^ in eq 4, as follows:
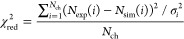
4where *N*_ch_ is the number of channels considered in the fit, *N*_exp_(*i*) and *N*_sim_(*i*) are the number of counts in channel *i* in the measured and simulated spectrum, respectively, and σ_*i*_ is the statistical error. The cost function minimization was carried out by a MATLAB implementation of a Nelder–Mead algorithm^40^ based, bounded minimization procedure.^41^ For this task, *N*_sim_, calculated by SIMNRA, was varied via changing the surface concentrations, the gradient values, and the shape correction factor by the minimizer routine, until a convergence was found.

**Figure 4:**
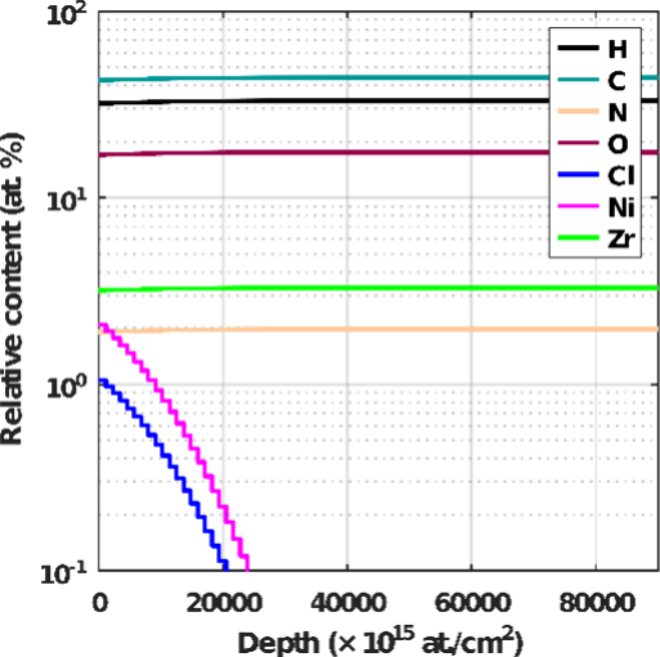
Relative atomic composition of the MOF crystal metalated with NiCl_2_ for 2 h, as a function of sample depth. Ni and Cl, as postsynthetically incorporated elements, feature a gradient according to eq 1, while all the other elements, being part of the initial MOF crystallite, maintain a constant ratio among themselves according to Table 1.

**Figure 5:**
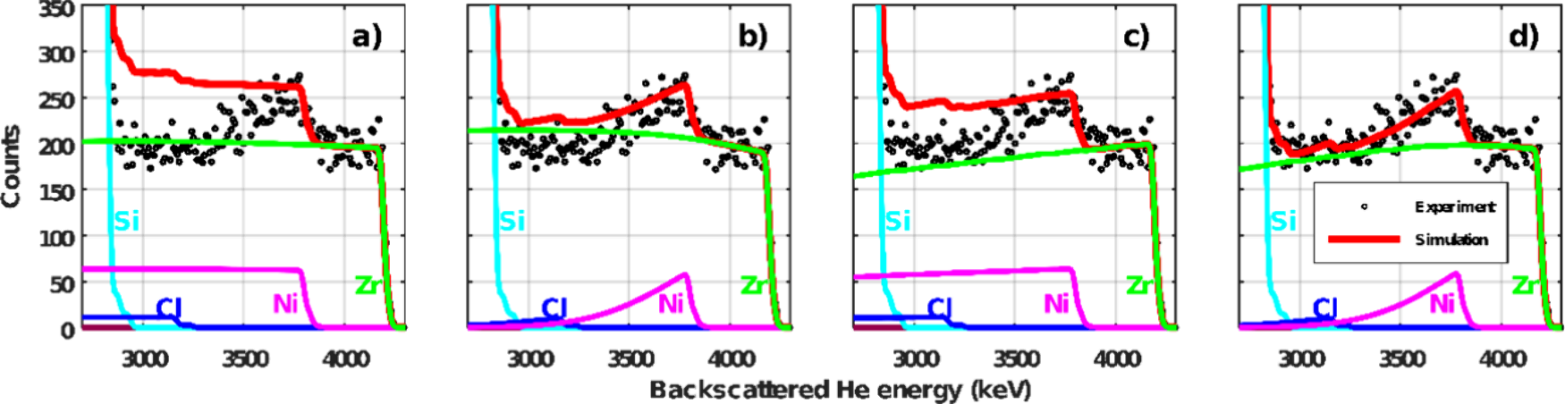
μ-RBS spectrum, showing the Zr, Ni and Cl region above the substrate signal, of the UiO-67-bpy MOF crystal metalated with NiCl_2_ for 2 h and fits according to four different scenarios. (a) No gradient of Ni and Cl was observed, and no shape correction is applied. (b) Assumed gradient of Ni and Cl, but no shape correction is applied. (c) No gradient in Ni and Cl, but shape correction is applied. (d) Assumed gradient in Ni and Cl, and shape correction is applied.

As a representative example, Figure 4 shows the fitted composition of the RBS spectrum of the MOF sample metalated for 2 h. It is visible in the figure that Ni and Cl feature a strong gradient in concentration, while the base crystal elements, including the organic and inorganic building units, maintain a constant ratio throughout the full crystal thickness. The RBS spectrum, highlighting the signal of Zr, Ni and Cl on the top ca. 6 μm (i.e., where they are not suppressed by the signal of the substrate and the lighter elements), and the fit of this spectrum are shown in Figure 5. This figure illustrates the sequence of how the different features of our model, namely (i) the elemental depth gradients, (ii) the shape correction, and (iii) their synergy, contribute to finding an overall composition that best describes the MOF sample. It is easily detectable that the best result is obtained when the model features both the true element gradients and the virtual gradients due to the crystal shape.

The surface concentrations, the characteristic diffusion lengths, and the shape correction factors as a result of the RBS spectrum fits are summarized in Table 2. In addition, diffusion coefficients are calculated from the fit results using eq 2. For the conversion from areal density (i.e., atoms/cm^2^, the commonly used thickness unit in Rutherford backscattering spectrometry) to SI units, the density of the material was estimated from the weighed average of the densities of individual constituents.

**Table 2:** Summary of the Results: Surface Ni Concentrations, Characteristic Diffusion Lengths, Shape Correction Factors As a Result of the Fits of the Measured RBS Spectra, and Diffusion Coefficients Calculated from the Fit Results

metalation time (h)	Ni surface conc. (atom %)	diffusion length(×10^15^atom/cm^2^)	shape correction factor	diffusion coeff. (m^2^s^–1^)
2	1.96	8307	0.47	1.60 × 10^–16^
24	1.98	31,622	0.48	1.93 × 10^–16^

We note that although Fickian diffusion implies an independent relation between crystal size and diffusivity, we use a 1D-layered structure in the SIMNRA model, which is therefore affected by the crystal size. A systematic error is imposed on the results, which might become significant in the case of small crystals, i.e., when the vertical projection (that is perpendicular to the incident beam) of the material diffusing from the sides occupies a large area compared to the entire crystal. In the present case, measurements on MOFs with different sizes did not show any correlation between the size and calculated diffusivity.

### Lateral Profiling

Particle-induced X-ray emission (PIXE) analysis has a larger information depth than RBS for a given incident beam. This is because the attenuation of the generated X-rays in a light matrix, such as the UiO-67-bpy MOF crystal, is small enough so that the generated X-rays can easily escape from sample depths where the RBS signal of the heavy elements starts to overlap with the signal of the light elements that are near-surface. Here, we present a case where the postsynthetic metalation has progressed deep enough into the crystal for the RBS data to suggest a nearly uniform Ni incorporation. However, the lateral Ni concentration profile extracted from the μ-PIXE measurement indicates a weak but evident gradient.

First, in order to obtain an estimated metalation time needed to reach such circumstances, we performed finite difference calculations solving the diffusion equation, , where *c*(*x,y,z,t*) is the time- and space-dependent concentration, *t* is the elapsed time, and *D* is the diffusion coefficient. Since the aim was not to obtain highly accurate information, we used a simplified model considering a rectangular domain. However, to keep the model as accurate as possible,

1. it was scaled to the approximate size of the MOF crystals used in the experiments (side length *l* = 20 μm and thickness *t* = 10 μm),
2. the diffusion coefficient (*D* = 1.6 × 10^–16^m^2^s^–1^) obtained from the first RBS measurement was used, and
3. the boundary conditions were set according to the experiments, i.e., zero-flux at the bottom plane, assuming prohibited NiCl_2_ transfer from the MOF to the Si substrate, and constant (unit) value at the sides and on the top, assuming constant solution concentration during metalation.

**Figure 6:**
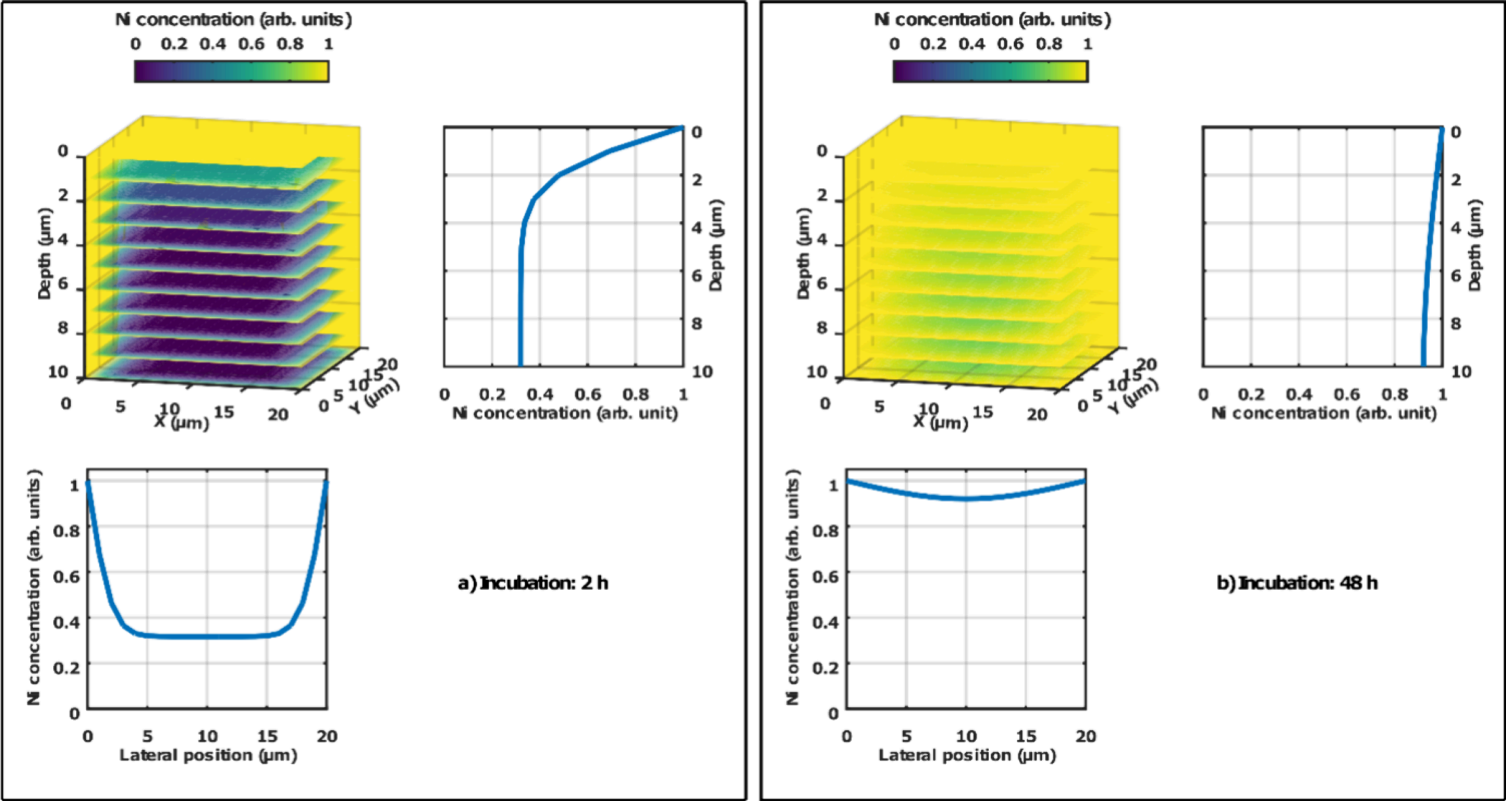
Finite difference simulation results of the expected Ni distribution in the MOF crystals and the corresponding depth profile (representative of depth profiling by μ-RBS) and lateral profile (representative of lateral profiling by μ-PIXE) in the case of (a) 2 h incubation time and (b) 48 h incubation time.

This model was precise enough to find a rough estimate on the metalation time necessary to reach a state where the lateral element concentration profile is still significant, while the depth profile on the top 6 μm (approximate depth before the signal originating from the Si substrate starts to overlap with the Zr and Ni signals) is already not. The results of the simulations are illustrated in Figure 6. After 2 h of incubation (Figure 6a) the concentration of Ni at a depth of 6 μm is already approaching its asymptote, which should be easily detectable in the RBS spectrum (note that the asymptote is around 0.3 units due to the finite grid size). Furthermore, the lateral concentration profile also shows a large contrast between the edges and the center region of the crystal. These observations are in line with the experiments (here, we refer back to Figure 2, where the RBS spectrum indicated a Ni depth gradient and the Ni PIXE element map indicated higher concentrations near the edges). In contrast to the short metalation time, the simulation shows that after 48 h of incubation (Figure 6b) the Ni concentration at 6 μm depth drops to only around 93% of the surface concentration. Considering that the surface concentration for Ni is only 2%, this is equivalent to such a small gradient that is likely not possible to identify in the RBS spectrum within the statistical uncertainty of the spectrum, given that the Ni signal originating from this depth is superimposed on the Zr and the surface Cl signal. However, after 48 h metalation, the μ-PIXE profile (Figure 6b) shows a relative concentration difference of around 90% between the center and the edges. Since the signals in the PIXE spectrum of the MOF are not superimposed and have better statistics, its significance is pronounced and we expect this lateral concentration gradient to be detectable in the measurements.

**Figure 7:**
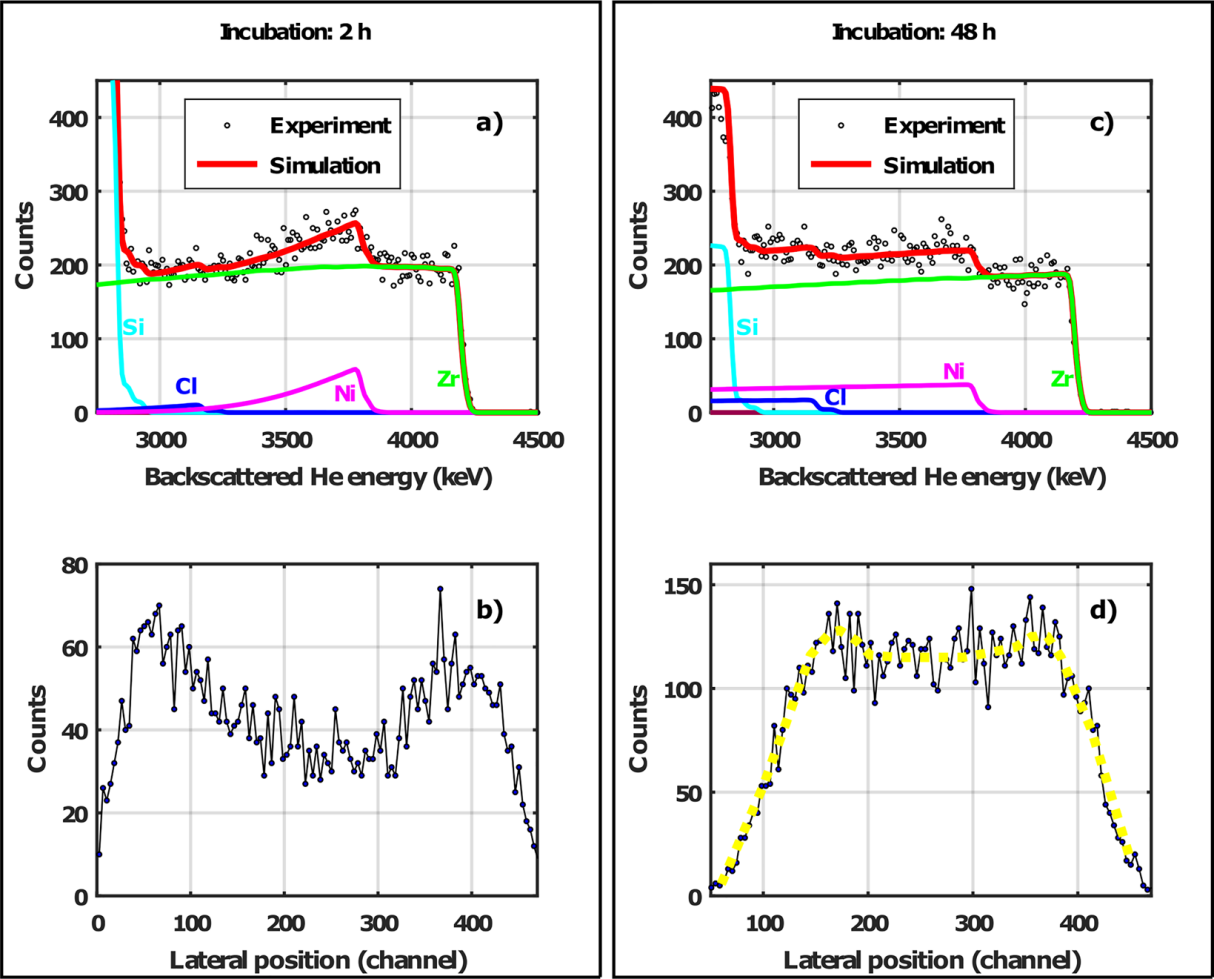
(a) μ-RBS spectrum for the Zr, Ni and Cl signal on the top ≈6 μm of a MOF single crystal metalated for 2 h. (b) Lateral profile of the Ni μ-PIXE signal of the same MOF crystal. (c) μ-RBS spectrum of a MOF single crystal metalated for 48 h. (d) Lateral profile of the Ni μ-PIXE signal of the same MOF crystal. The yellow dashed line guides the eye to a better view of the lateral profile.

According to the results of the simulation, a new UiO-67-bpy MOF sample was prepared and metalated for 48 h. The sample was then measured under the same conditions as the ones metalated for 2 and 24 h using a 5 MeV He^2+^ microbeam. The measured RBS spectrum and lateral PIXE profile for Ni are illustrated in Figure 7, together with the sample that was metalated for 2 h, for direct comparison. Although quantitatively different, the experimental results qualitatively match the simulations. After 48 h of metalation, the RBS spectrum shows nearly uniformly distributed Ni on the top 6 μm: the slope of the Ni signal is practically equal to that of the Zr signal. However, the lateral profile for the Ni PIXE signal indicates a slight but significant contrast in the Ni concentration between the edge and center regions.

The observations presented in this subsection outline another aspect of our approach: by varying the metalation time, the range of detectable diffusion coefficients is extensible. In the case of fast diffusion, short metalation time will result in a distribution that can be accurately quantified by μ-RBS, while a very slow diffusion process can result in an easily detectable gradient in the RBS spectrum after accordingly long metalation time.

### Long-Term Stability of the Postsynthetically Metalated UiO-67-bpy MOFs

**Figure 8:**
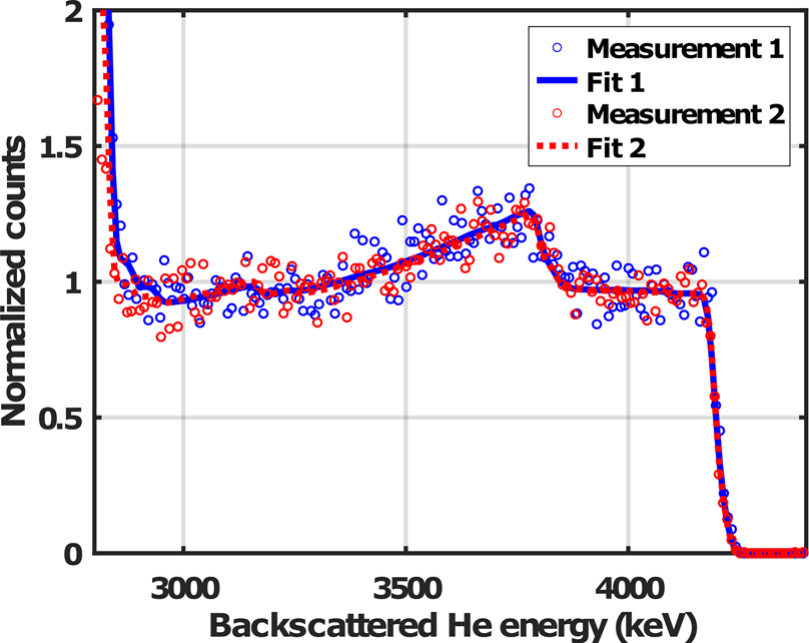
μ-RBS spectra and corresponding SIMNRA fits of two different UiO-67-bpy single crystals from the same sample, measured with a 12 months difference.

The long-term stability of MOFs is a critical factor in practical applications.^42^ This statement usually concerns the physical (lattice) structure, but the chemical composition is also important from technological aspects. Stability might be an issue also for analysis techniques employing large-scale facilities, such as ion-beam (micro)analysis, because of the typically long lead time. In this subsection, we present the results of a repeated μ-RBS measurement on the sample that was metalated for 2 h, carried out with more than 12 months difference. For the repeated measurements, another individual MOF crystallite was chosen from the same sample. Between the two measurements, the sample was stored in air at room temperature. Figure 8 shows the RBS spectrum of the measurement 12 months after synthesis and the result of the fitting procedure together with the initial spectrum and its fit for direct comparison. From this figure it can be seen that the repeated measurement yielded very similar results: the measured surface concentration for Ni is 2.08 atom %, and the calculated diffusion coefficient is 1.63 × 10^–16^ m^2^s^–1^, in comparison to the 1.96 atom % surface Ni concentration and 1.60 m^2^s^–1^ diffusion coefficient obtained in the first measurement. From these results, we conclude that the chemical composition of the UiO-67-bpy MOFs proved to be stable during storage in air at room temperature, on a year-long time scale.

Given that the repeated measurement yielded very similar values which did not exceed the expected statistical uncertainty of our measurement, we calculated an overall diffusion coefficient for all measurements including the 2 h, the 24 h, and the repeated 2 h metalation ones. The calculated average diffusion coefficient of NiCl_2_ in the UiO-67-bpy MOFs under the specific metalation conditions is (1.72 ± 0.18) × 10^–16^ m^2^s^–1^.

### Potential Complementary Techniques

In this section, we discuss a few other techniques that could potentially be used to assess the metal incorporation into MOF crystals.

Scanning electron microscopy combined with energy-dispersive X-ray spectroscopy (SEM–EDX) is a widely used technique, owing to the relatively small instrumentation needed. SEM–EDX provides fast elemental mapping without the need for special sample preparation. The spatial resolution is on the order of 10 nm, and the probing depth is on the order of 1 μm, which makes it well suited for smaller crystal sizes or large crystals where the metal penetration is not too deep. SEM–EDX would provide a relatively simple platform to qualitatively assess the depth distribution of the elements in MOFs.

Nuclear magnetic resonance (NMR) is a quantitative technique that is capable of probing molecular motions, including translational diffusion in pulsed-field gradient mode, on the microscopic scale.^43^ It can be used to determine molecular diffusivities in a wide range, and it has been extensively applied to MOF structures, in particular, to study the diffusion of hydrocarbons (e.g., ref (44)). In the present case, ^61^Ni could be directly probed by NMR, but the technique could be applied to other materials as well.

Finally, hard X-ray photoelectron spectroscopy (HAXPES) provides information about the electronic structure and the chemical states down to a few 10s of nanometers. By varying the photon energy, we could directly obtain depth profiles in this depth range, but alternatively, at a fixed photon energy, a time-resolved measurement could also be used to probe the diffusion properties of the guest material in MOF crystals.

In summary, several analysis techniques exist that can be used to qualitatively/quantitatively assess the depth distribution of postsynthetically incorporated elements in MOFs, and μ-RBS and μ-PIXE, featuring their own advantages and disadvantages, undoubtedly enrich the repertoire of available methods for the MOF community.

## Summary and Outlook

Our proposed method, based on goodness-of-fit minimization of μ-RBS spectra, provides quantitative values of composition gradients along depth for selected elements in highly complex systems, such as MOFs, in a nondestructive manner. As it was presented in this work, elements that are heavier than the most abundant elements of the sample, even if not the heaviest and their surface concentration is a few atomic percent at most, can be accurately depth-profiled by RBS, potentially down to several micrometers. The data extracted from the depth profiles show that the postsynthetic metalation of UiO-67-bpy (30% bpy-based linker) MOFs with NiCl_2_ at 100 °C is a diffusion-limited process and the diffusivity of the investigated system is (1.72 ± 0.18) × 10^–16^ m^2^s^–1^. When the information depth of μ-RBS is insufficient to obtain statistically significant results, lateral concentration profiles from simultaneous micro-PIXE (μ-PIXE) analysis can still indicate indirectly the presence of depth gradients through an edge-effect, i.e., when the element concentration decreases toward the core of the crystal due to diffusion from the outer surfaces. The long-term stability of the spatial distribution of elements incorporated into the UiO-67-bpy MOF was proven by repeated measurement.

The method presented herein is universally applicable, providing the possibility of investigating the transport process of other metals or other MOF systems as well. As an example, preferential incorporation in mixed metal systems might be revealed and quantified analogously. Furthermore, spatially resolved, element-sensitive analysis of MOF structures by MeV ion microbeams holds a large potential through the diversity of available techniques. The range of elements detected by PIXE analysis can be potentially extended down to carbon, although with a limited depth range for Na and lighter elements due to the large self-attenuation of their low-energy X-rays. Light elements could be detected, isotope-specifically using particle-induced gamma emission (PIGE) analysis using a proton/deuteron beam, analogously (and also simultaneously) to the PIXE measurement presented herein but extending the Z-range down to Li. Furthermore, with nuclear reaction analysis (NRA) hydrogen becomes detectable with fine depth resolution, or after isotope labeling, deuterium can be probed down to several micrometers.

## Supporting Information Available

Optical microscope images; measured and simulated XRD spectra; and NMR spectrum (PDF)
