# Supplementary material for: Evaluating Nondestructive Quantification of Composition Gradients in Metal–Organic Frameworks by MeV Ion Microbeam Analysis
Source: Anal Chem. 2024 Sep 10;96(38):15285–94. doi: 10.1021/acs.analchem.4c02730 (PMC11428091; doi:10.1021/acs.analchem.4c02730)
Supplement: Supplementary file 1 — ac4c02730_si_001.pdf [file ac4c02730_si_001.pdf]

Supporting Information

Evaluating non-destructive quantification of composition gradients in metal-organic frameworks by MeV ion microbeam analysis

Gyula Nagy[1,*], Wanja Gschwind[2], Sascha Ott[2], and Daniel Primetzhofer[1]

[1]Department of Physics and Astronomy, Uppsala University, SE-751 20 Uppsala, Sweden

[2]Department of Chemistry, Uppsala University, SE-751 20 Uppsala, Sweden

*Corresponding author. Email: gyula.nagy@physics.uu.se

Contents

Figure S1-2. Optical microscope
Figure S3. XRD pattern
Figure S4. NMR spectrum

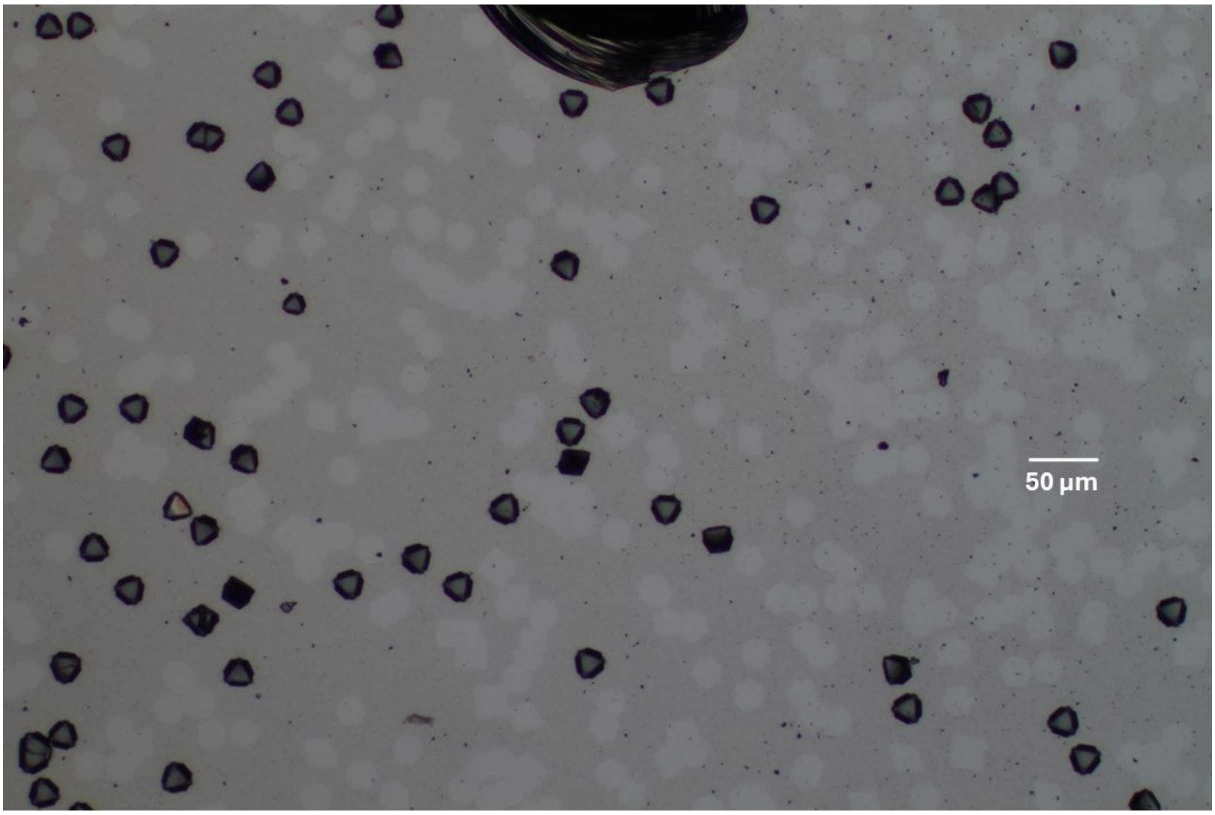

Figure S1. Typical optical microscope picture of UiO-67 MOF single crystals on Si substrate.

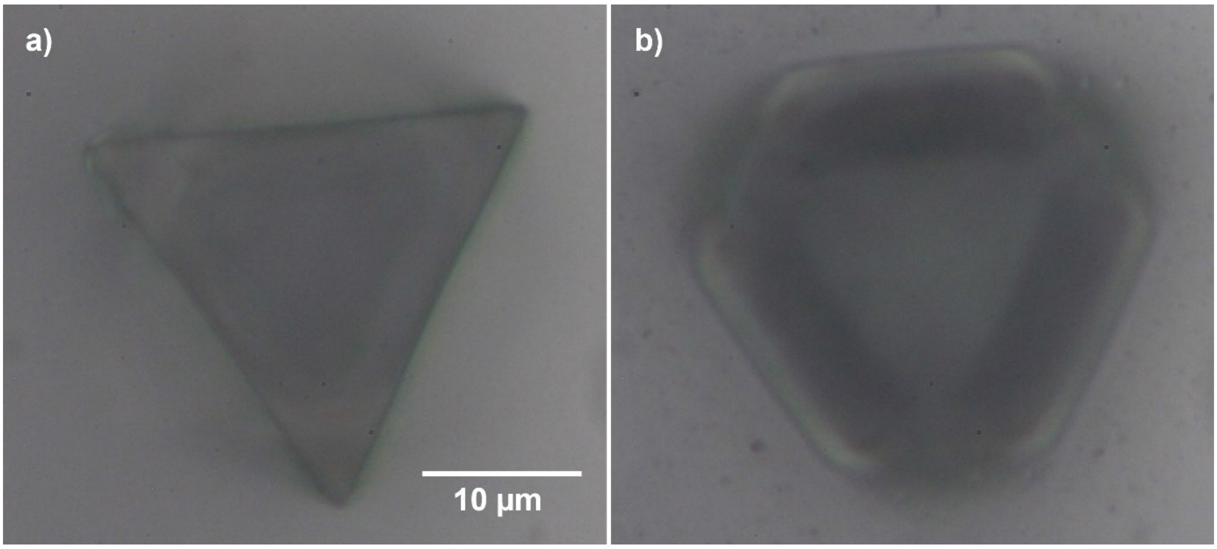

Figure S2. Optical microscope picture of an individual MOF crystal used for this work (24 h metalation). **a)** Focus on top plane. **b)** Focus on bottom (substrate) plane.

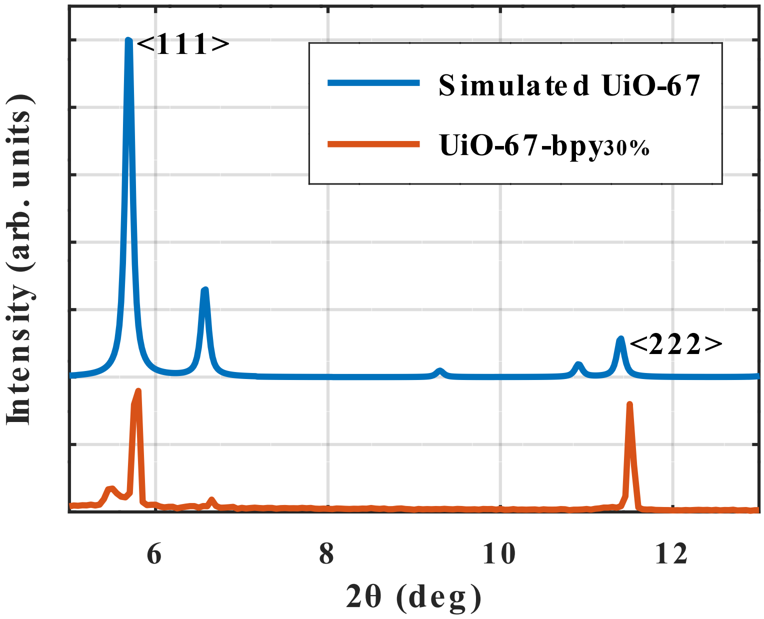

Figure S3. XRD pattern measured prior to metalation (red), and simulated XRD pattern of bulk UiO-67 (blue).

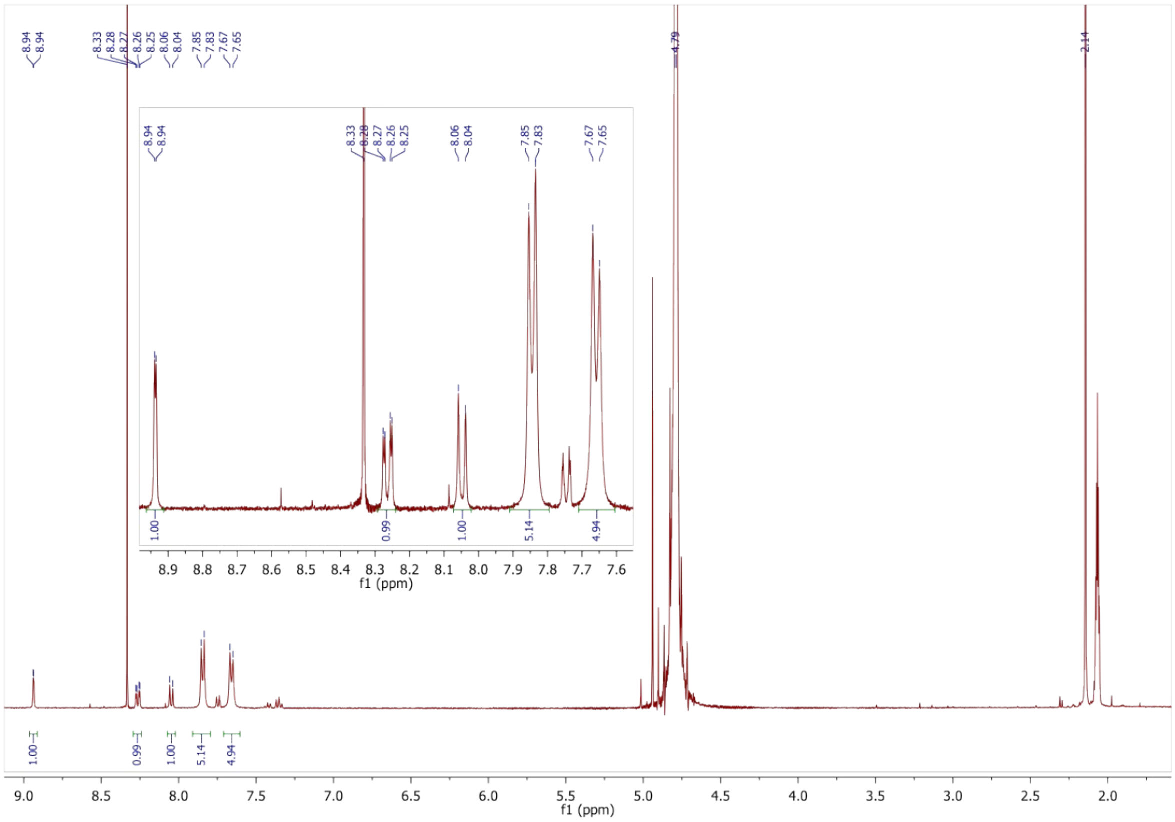

Figure S4. NMR spectrum of a digested MOF sample (before metalation). The calculated bpy-to-bpdc ratio is 30 ± 3 %.
